# Invited discussant comments during the UCL–Penn Global COVID Study webinar ‘How Do We Trust (Again): Paranoia and Mental Health’: part 1 of 2

**DOI:** 10.14324/111.444/ucloe.100002

**Published:** 2022-11-01

**Authors:** Emma Barkus

**Affiliations:** 1 Department of Psychology, Northumbria University, Newcastle upon Tyne, UK

**Keywords:** schizotypy, anxiety, depression, stress, loneliness, sleep, Covid-19, mental health, longitudinal, health, social value, community consultation, design interventions, green infrastructure, multi-functional benefits, stakeholders

## Abstract

The article provides commentary on Wong et al.’s investigation of the relationship between schizotypal traits, social mistrust and aggression, mental and physical health outcomes across three waves of data collection commencing in April 2020. The researchers aimed to consider the nature of the relationship between these variables and the stability of these relationships as coronavirus (Covid-19) restrictions fluctuated over time. Their results suggested that loneliness reflects a hub which links the trait variables of schizotypal and social mistrust to aggression and mental and physical health symptoms. Their network did not vary by demographic factors nor wave of data collection, suggesting that stable individual differences were driving results. Their results propose that interventions which increase social connection could provide positive health benefits as well as decreasing aggression (via reductions in social mistrust). Their data contributes to understanding about how schizotypal traits link to outcomes under conditions of social stress.

**About the study**

The UCL–Penn Global COVID Study^1^, launched in April 2020, is a 12-month longitudinal study of the impact of Covid-19 on social trust, mental health and physical health. In collaboration with six institutions from Italy, Singapore, the United States, China and the United Kingdom,^2^ the study looks at the short- and longer-term effects of Covid-19 on individuals’ mental health and social relationships with others. Survey data were collected at three time-points: 17 April–14 July 2020 (Wave 1), 17 October 2020–31 January 2021 (Wave 2) and 17 April–31 July 2021 (Wave 3).

**About the webinar**

Held online between 2 June and 28 July 2021, the study group presented research data at five online webinars, as part of the UCL Global Engagement Fund sponsorship, to discuss the lessons learned, and invited policy makers and other subject experts to speak on the policy relevance and implications of the study findings. The recorded comments from these discussions, focusing on the policy relevance and implications of each academic article, were recorded as discussant articles and are published in this journal to be read alongside the research article being discussed.

These discussant articles are reviewed by members of the Editorial Board before being published. It is hoped that these discussant articles, read alongside the academic articles, will provide a more holistic understanding of the issues at hand, how findings may inform policies in the coming months and/or assist in future crisis management strategies and aid decision-making, in an open and transparent manner.

The study was pre-registered https://osf.io/4nj3g/ on 17 May 2021) and ethical approval was obtained from the IOE (Institute of Education), UCL’s Faculty of Education and Society (University College London, UK) Ethics and Review Committee on 8 April 2020 (REC 1331).^1^

**Linked research article**

The linked research article to this discussion article cited here has been published in *UCL Open: Environment* following open peer review and made freely available to read as an open access article. Additionally, all previous versions and peer review reports are freely available to read as open access preprint articles from the journal’s preprint server by following the below DOI link and navigating to the version history of the published research article. Readers can find more information about how peer review works in the journal at ucl.scienceopen.com.

Wong KK, Wang Y, Esposito G, Raine A. A three-timepoint network analysis of Covid-19’s impact on schizotypal traits, paranoia and mental health through loneliness. *UCL Open: Environment*. 2022;(4):17. Available from: https://doi.org/10.14324/111.444/ucloe.000044

**Recorded webinar**

This discussion article comments on the findings presented during the following webinar that has been recorded and made freely available to readers to watch on-demand.

Summer Webinar 2 – Social Trust & Mental Health #GlobalCOVIDStudy. Available from: https://www.youtube.com/watch?v=AcQO1owUNFA

## Introduction

The latter part of 2019 and the start of 2020 marked a fire season in Australia of a ferosity that has not been seen since the ‘Black Saturday’ events in Victoria, Australia in 2009. People were no longer safe in their own homes; wildlife, tracts of bushland, homes, business and lives were lost in the face of a fire front which, in many instances, was uncontrollable, inducing fear in those indirectly and directly concerned. Communities rallied: clothed, fed, protected and sheltered those affected with open arms of concern, cohesion and collective support. People came together to face a highly visible fear. While there may have been uncertainty regarding the fires’ movements, people were certain in what they could do to help others and protect themselves.

While Australia was preoccupied by fires, over December 2019 and early January 2020 there was a hidden nemesis stealthily creeping its way across our globe through our international cooperation, trade and travel routes: coronavirus (Covid-19). Globalisation and the free movement of humans within our world actively worked against humanity in the spread of Covid-19. In comparison to a fire, Covid-19 is an unseen threat which spread through the very communities from which support was previously so crucial. Inevitably, most countries have experienced periods where social isolation, distance or separation was necessary, with people staying within their homes: the only safe place. The walls of home became a barrier for safety, with friends, foes, strangers and acquaintances all equally a potential Covid-19 threat. Rather than bringing people together to fight a common enemy, people were being driven apart both physically and psychologically. Families were divided over how conservatively regulations and guidelines should be followed; attitudes and beliefs about vaccination are still proving divisive. Evidence exists from other large-scale events to suggest an increase in both mental health disorders and subclinical symptoms follows in their wake. For example, in a cohort study, exposure to four major earthquakes over a 12-month period in Canterbury, New Zealand was associated with a 1.4% increased rate of mental health disorders or subclinical symptoms [1]. Unsurprisingly, over the Covid-19 pandemic there has been worldwide increased depression and anxiety [2], prevalence of mental health disorders in healthcare workers [3] and in people shielding or who contract Covid-19 [4]. This suggests some groups within our communities may be more vulnerable to mental health outcomes.

Given the general level of distrust within communities, it is unsurprising that paranoia, social mistrust and negative attitudes towards others have increased internationally during the unfolding of the Covid-19 pandemic [5,6]. How information is delivered during a crisis has also been reported to predict distress: for example, university students who received and believed conflicting information (via social media) while in a lockdown following a shooting incident on campus, reported increased distress [7]. Fear of misinformation and fake news have been reported as a community concern during the Covid-19 pandemic [5]. Collectively, these studies raise questions about whether there may be individual characteristics or traits which leave people vulnerable to paranoia and social mistrust in response to the circumstances surrounding the Covid-19 pandemic.

Keri Wong and Wang Yi presented data from the UCL–Penn Global COVID Study during a webinar held on 16 June 2021. Wong et al. wished to investigate whether schizotypal traits and social mistrust were associated with aggression and mental and physical health symptoms across three waves of data collection in different countries around the world collected in April 2020, and 6 months and 12 months after this date. Schizotypal traits reflect a collection of personality factors comprising attenuated forms of behaviours, thoughts and experiences reported in people with *Diagnostic and Statistical Manual of Mental Disorders*, 5^th^ Edition (DSM-5) Psychotic Disorders. This constellation of personality traits appears to leave people from the general population vulnerable to common mental health symptoms as well as unusual thoughts and perceptual experiences. Wong et al. utilised a statistical method called network analysis to consider the links between the schizotypal traits, loneliness, social mistrust, aggression, generalised anxiety symptoms, Covid-19-related stress, physical health and sleep. Understanding how these factors relate to one another could provide clues to particularly sensitive points for positive change by future targeted interventions.

## Discussant comments

The network analysis of the Wave 1 data revealed strong links between the three schizotypal traits. Interpersonal schizotypal traits were the most strongly linked dimension to loneliness and loneliness acted as a hub connecting schizotypal traits to mental and physical health symptoms. Of the schizotypal dimensions, Cognitive Perceptual was most strongly linked to social mistrust, with a weaker link between Disorganised traits and social mistrust. The interlink between schizotypal and social mistrust traits forms a web of personality characteristics which could drive social disconnection through devaluing social connections, increasing social anxiety and mistrust, as well as laying the foundations for cognitive biases which exacerbate tendencies for social withdrawal. While evolutionary theories of loneliness suggest loneliness exists as an aversive state to encourage people to seek out and re-establish social connection, it is possible that the characteristics captured by schizotypal and social mistrust traits lay the foundations for cognitive barriers to perceive social environment as threatening and/or unbeneficial.

Social mistrust and Cognitive Perceptual connect the schizotypal traits web to aggressive tendencies. Paranoia has been associated with both low [8,9] or increased [10] adherence to Covid-19 safety guidelines in other studies. It may be that beyond a certain threshold, the presence of mistrust and paranoia underpins behaviours which exacerbate, rather than alleviate, people’s fear and anxiety. In speculating, this fear and anxiety may then drive escalating patterns of thought, which isolate people reducing the likelihood that reality checking or hypothesis testing can take place to thwart the processes underpinning paranoia and mistrust. This creates a vicious circle of behaviour and cognitions which drives maladaptive emotions.

The network did not vary by age, gender, country nor income groups; in addition, the network pattern between variables did not vary by wave of data collection. Previous studies have suggested that while there is variation in how people respond to a big event when it occurs, ongoing life stressors following the event (related or unrelated) during the course of people’s lives contribute most significantly to continued mental health disorders in the subsequent years [11]. This reflects that people’s tendencies, traits or vulnerabilities, interact with events that occur in their lives to enable mental health difficulties to surface, that is, the environment acts as a potentiator to expose people’s predispositions to mental and physical health symptoms [12]. Previous literature suggests schizotypal traits predict meaningful variations in people’s experiences in the flow of everyday life [13]. Between April and October 2020, when Wong et al. were gathering their data, there were multiple changes in Covid-19 restrictions. Compared to Wave 1, at Wave 2, participants had lower levels of aggression and more sleep problems; while lower schizotypal traits and Covid-19 stress were reported at the final wave of data collection. However, the relationship between the variables remained consistent, suggesting individual stable factors are driving findings. In predicting future emotions, people are more accurate at predicting intensity of emotions than intrusiveness and mood [14]. The intensity of people’s responses to an event are likely driven by personality traits; therefore people are able to anticipate how they will respond immediately to an event, but not how preoccupied they will be by the event, nor how much day-to-day concerns will evolve at the same time to occupy their emotions and thoughts. The networks varied with higher levels of schizotypal traits and social mistrust such that links between these and loneliness were amplified; suggesting that loneliness acts as a social stressor to link schizotypal traits and social mistrust to mental and physical health symptoms [15].

## Summary

The network analysis presented by Wong et al. suggests loneliness is a pivotal factor linking stable personality vulnerabilities to aggression, mental and physical health symptoms. Loneliness can be viewed as a social stressor which triggers physiological responses which are deleterious for health [15]. While loneliness links the schizotypal web to mental and physical health symptoms, social mistrust provided the link to aggressive tendencies. Previous literature has suggested victimisation or peer relations are involved in the relationship between schizotypal traits and aggression [16–18]; social mistrust could act as the stable characteristic which underpins the interpretation of interpersonal difficulties. Loneliness comprises both state and trait elements: people may have a stable tendency towards feeling lonely, while certain circumstances may induce feelings of loneliness which are transient. Adding to the complexity, it is necessary to have a degree of social mistrust to ensure that we do not trust without healthy concerns or boundaries. Therefore, both loneliness and social mistrust reflect factors which intensify how characteristics like schizotypal traits interact with external circumstances, such as the Covid-19 pandemic, to predict multiple behavioural and health outcomes. The data presented by Wong et al. suggests loneliness interventions which increase prosocial feelings present an opportunity to garner positive health outcomes. One common path for reducing both aggression and loneliness could be prosocial behaviour [19]. The messaging and government responses to Covid-19 have created uncertain and ambiguous circumstances, open to multiple interpretations. People expressing schizotypal traits may be particularly vulnerable to unhelpful perceptions of their environment, ensuring that they withdraw, feel suspicious and disconnected from others. The results from Wong et al.’s study suggest that to improve people’s longer-term psychological and physical health outcomes, as Covid-19 decreases as a threat, public health messaging and interventions need to increase social connections and positive interpretations of others. Wong et al. are providing clear points for future interventions which will benefit people’s health and decrease aggression, improving social environments as well as health.

## Notes

1 Wong KK, Raine A. Covid-19: Global study of social trust and mental health. Available from: https://globalcovidstudy.com [Accessed: 11 November 2021].

2 Wong KK, Raine A. Covid19: Global social trust and mental health study. 2020. https://doi.org/10.17605/OSF.IO/FE8Q7.
